# Developing A Smartphone-Based Application for the Behavioral Cognitive Therapy of Panic Disorder

**DOI:** 10.1192/j.eurpsy.2022.1469

**Published:** 2022-09-01

**Authors:** E.C. Esen, H. Baykan

**Affiliations:** 1 Balikesir Universitesi Egitim ve Arastirma Hastanesi 1.Kat Psikiyatri AD., Altieylul/ Balikesir, Balikesir, Turkey; 2 Balikesir University, Department Of Psychiatry, Balikesir, Turkey

**Keywords:** e-mental health, panic disorder, mobile application

## Abstract

**Introduction:**

Even though cognitive behavior therapy is proven to be an effective treatment for panic disorder, the scarcity of psychiatrists cause many patients not to get a sufficient therapy. E-mental health applications are being developed to address this shortage, especially after the COVID-19 pandemic. However, none of the e-mental health applications developed so far has offered a structured cognitive behavioral therapy.

**Objectives:**

We are developing a mobile application which will integrate with psychiatric interventions that aims to make cognitive behavioral therapy more accessible.

**Methods:**

Our algorithm consists of multiple choice questions and answers to determine the progression of the algorithm. The first three sessions consist of psycho-education of the application and the cognitive therapy model of panic mostly. During the psycho-education sessions, patients’ symptoms during panic attacks and their catastrophic thoughts will be questioned to be used in following sessions. After the panic log has been introduced in the third session, patients will enter the details of their panic attacks right after they experience it and this information will be investigated in the following sessions. Progress for the cognitive restructuring will be monitored as the sessions proceed. Later session will also include in-session symptom induction exercises.

**Results:**

We are still on the development phase of the mobile application. Hence we do not have any data to present at the moment.

**Conclusions:**

Our main purpose is to develop a mobile application which will integrate with structured cognitive behavioral therapy process, reduce the workload of the therapist and is easily accessible through the smart phones.

**Disclosure:**

No significant relationships.

